# Presence of contractile impairment appears crucial for structural remodeling in idiopathic left bundle-branch block

**DOI:** 10.1186/s12968-021-00731-6

**Published:** 2021-04-01

**Authors:** Janek Salatzki, Theresa Fischer, Johannes Riffel, Florian André, Kristóf Hirschberg, Andreas Ochs, Hauke Hund, Matthias Müller-Hennessen, Evangelos Giannitsis, Matthias G. Friedrich, Eberhard Scholz, Norbert Frey, Hugo A. Katus, Marco Ochs

**Affiliations:** 1 Department of Cardiology, Angiology and Pneumology, Heidelberg University Hospital, Im Neuenheimer Feld 410, 69120 Heidelberg, Germany; 2 DZHK (German Centre for Cardiovascular Research), Partner site Heidelberg, Heidelberg, Germany; 3 Semmelweis University Heart and Vascular Center, Budapest, Hungary; 4 Division of Cardiology, Departments of Medicine and Diagnostic Radiology, Mc-Gill University Health Centre, Montreal, Canada

**Keywords:** Left bundle-branch block, Septal flash volume, Fibrosis, Remodeling

## Abstract

**Background:**

To differentiate effects of ventricular asynchrony from an underlying hypocontractile cardiomyopathy this study aimed to enhance the understanding of functional impairment and structural remodeling in idiopathic left bundle-branch block (LBBB).

We hypothesize, that functional asynchrony with septal flash volume effects alone might not entirely explain the degree of functional impairment. Hence, we suggest the presence of a superimposed contractile cardiomyopathy.

**Methods:**

In this retrospective study, 53 patients with idiopathic LBBB were identified and matched to controls with and without cardiovascular risk factors. Cardiovascular magnetic resonance (CMR) was used to evaluate cardiac function, volumes and myocardial fibrosis using native T1 mapping and late gadolinium enhancement (LGE). Septal flash volume was assessed by CMR volumetric measurements and allowed to stratify patients with systolic dysfunction solely due to isolated ventricular asynchrony or superimposed contractile impairment.

**Results:**

Reduced systolic LV-function, increased LV-volumes and septal myocardial fibrosis were found in patients with idiopathic LBBB compared to healthy controls. LV-volumes increased and systolic LV-function declined with prolonged QRS duration. Fibrosis was typically located at the right ventricular insertion points. Subgroups with superimposed contractile impairment appeared with pronounced LV dilation and increased fibrotic remodeling compared to individuals with isolated ventricular asynchrony.

**Conclusions:**

The presence of superimposed contractile impairment in idiopathic LBBB is crucial to identify patients with enhanced structural remodeling. This finding suggests an underlying cardiomyopathy. Future studies are needed to assess a possible prognostic impact of this entity and the development of heart failure.

*Trial registration:* This study was retrospectively registered.

**Supplementary Information:**

The online version contains supplementary material available at 10.1186/s12968-021-00731-6.

## Background

Left bundle-branch block (LBBB) is considered to exaggerate or even cause ventricular dysfunction and myocardial remodeling resulting in increased mortality in patients with cardiovascular disease [1–3]. LBBB is usually associated with obstructive coronary artery disease (CAD), cardiomyopathy, hypertension or arrhythmia and the prevalence in the total population is estimated to be 1% [4, 5]. Heart failure patients with LBBB may benefit from cardiac resynchronization therapy (CRT) showing reverse remodeling with a recovery of left ventricular (LV) function [6, 7]. Due to changes in the electrical activation sequence LBBB results in ventricular conduction delay leading to irregular septal movement and the occurrence of septal flash volume [2, 8]. LBBB is considered as a hallmark of structural cardiac disease characterized by wall thickening, dilatation and reduced LV function [1, 3]. However, there are cases of idiopathic LBBB with reduced contractile function considered to be the consequence of dyssynchrony. Idiopathic LBBB is present in individuals without detectable cardiovascular disease and is described with a prevalence of 0.1% in the overall population [9, 10]. Recent imaging studies using echocardiography and cardiovascular magnetic resonance (CMR) in patients with idiopathic LBBB revealed reduced LV ejection fraction (LVEF) and increased LV volumes compared to healthy controls [1, 11]. However, it remains uncertain if dyssynchrony due to septal flash volume alone explains the degree of functional impairment or if the additional presence of a superimposed hypocontractile cardiomyopathy results in dysfunction and structural remodeling.

First, we hypothesized that ventricular conduction delay, expressed by the electrocardiogram (ECG) QRS duration, correlates with cardiac dysfunction, ventricular enlargement, myocardial fibrosis and septal flash volume. Second, we aimed to enhance the understanding of functional impairment in idiopathic LBBB and hypothesized that effects of asynchrony caused by septal flash volume and a contractile impairment are two different mechanisms contributing to structural remodeling in patients with idiopathic LBBB. To investigate the hypothesis, we used a novel method to calculate the septal flash volume in order to differentiate between LBBB patients with ventricular asynchrony and superimposed contractile impairment.

## Methods

### Study population and design

Potential patients with idiopathic LBBB were retrospectively identified from our local CMR and echocardiography database at the Department of Cardiology, Angiology and Pneumology, University Heidelberg during May 2009 and August 2019.

**Fig. 1:**
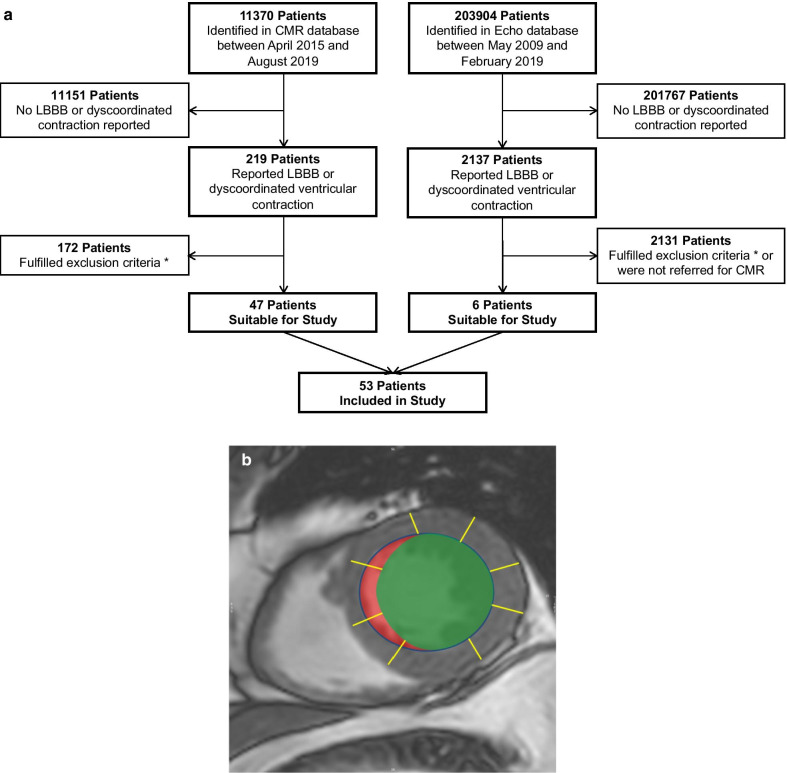
Flowchart of patient selection and assessment of septal flash volume.** a** Flowchart of patients selected for the study. *Exclusion criteria: greater than mild valvular disease, myocardial infarction, obstructive coronary artery disease (stenosis > 75%), positive stress test, cardiomyopathy, arrhythmia, prior cerebrovascular events and history of potential cardiotoxic chemotherapy exposure. CMR—cardiovascular magnetic resonance, LBBB—left bundle-branch block. **b** Representative image of assessment of flash volume using CMR short axis (SAx). Area within blue line: end-systolic volume, green area: corrected end-systolic volume by assuming a homogenous circumferential wall thickening, red area: flash volume

Patients with greater than mild valvular disease, myocardial infarction, obstructive CAD (major artery diameter stenosis > 75%), cardiomyopathy, arrhythmia, prior cerebrovascular events and history of potential cardiotoxic chemotherapy exposure were excluded (Fig. 1a). All patients had a low pre-test probability for CAD and either a negative stress test using CMR or echocardiography (n = 31) or an obstructive CAD was excluded using coronary angiography (n = 22).

LBBB has been confirmed by 12-lead ECG before or immediately after CMR. LBBB was defined as QRS duration > 120 ms as previously described [12]. The retrospective analyses of anonymized patient data was approved by the institutional ethics committee in accordance to the declaration of Helsinki (S-154/2015). The requirement for individual informed consent was waived by IRB. Cardiovascular disease (CVD) risk factors (arterial hypertension, hypercholesterinemia, diabetes mellitus, history of smoking and family history of CAD) and cardiac biomarkers (NT-proBNP and high-sensitive troponin T) were assessed using medical reports. Hypertension was defined as a systolic blood pressure ≥ 140 mmHg, diastolic blood pressure ≥ 90 mmHg or the use of antihypertensive medication.

### Selection of controls

Age-, sex-, body-surface area (BSA)- and CVD risk factor -matched individuals free of evident CVD or any conduction delay (control group A) were selected from our CMR database. These individuals were patients who underwent CMR due to a high risk for CAD or a family history of cardiomyopathy, but without any pathological findings on CMR. Exclusion criteria of controls were the same as for our study group.

Additionally, we compared our study group with healthy individuals without any CVD risk factors or history of heart disease (control group B). This cohort was also age-, sex-, BSA-matched.

### Study protocol

Standard CMR was performed supine in a CMR system (Achieva, 1.5 T, Ingenia 1.5 T, or 3 T Ingenia; Philips Healthcare, Best, The Netherlands), with a commercial cardiac phased array receiver coil as previously described [13, 14]. Details of acquisition and post-processing are available in the supplementary material. Global circumferential strain (GCS) and global longitudinal strain (GLS) were derived from cine balanced steady state free precession (bSSFP) of long axis 2-, 3- and 4-chamber and short axis views (SAx) by delineating LV endo- and epicardial borders at end-diastole using feature tracking technique. All post-processing measurements were performed with cvi^42^ software (version 5.6.6, Circle Cardiovascular Imaging Inc., Calgary, Alberta, Canada). CMR parameters were indexed to body-surface area.

Myocardial tissue characterization was assessed by measuring the longitudinal relaxation time constant of the myocardium (native myocardial T1 times) using a Modified Look-Locker Inversion recovery (MOLLI) sequence, 5 s(3 s)3 s variant in a midventricular SAx during breath-hold in end-expiration in the end-diastolic phase of the cardiac circle [15]. LV endocardial and epicardial borders were defined manually, using an offset of 10% to avoid partial-volume effects in the subendocardial and subepicardial layers using the cvi^42^ software (Circle Cardiovascular Imaging). Regional T1 times were measured using 16-segment American Heart Association model. T1 times of the segments antero-septal and infero-septal were combined and labelled “septal”, while segments antero-lateral and infero-lateral were combined and labelled “lateral”.”

T2 mapping was performed using multiecho Gradient-Spin-Echo (GraSE) sequence on the same ventricular short-axis slice as T1 mapping. Late gadolinium enhancement (LGE) images were acquired 10 min after administration of gadolinium diethylenetriamine pentaacetic acid /DTPA (Magnograf, Schering, Berlin, Germany) (before February 2016) or Gadobutol (Gadovist, Schering, Berlin, Germany) (after February 2016) and analysis was performed as previously described [16]. LGE was quantified using the cvi^42^ (Circle Cardiovascular Imaging) software and defined as areas with a signal intensity more than 5 standard deviations higher than the mean signal intensity of remote myocardium in the same short-axis Sect. [16]. Details of mapping and LGE acquisition are available in the supplementary material.

### Assessment of septal flash volume

By assuming a homogenous circumferential wall thickening, the corrected LV end-systolic volume (corrected ESV) was assessed. Hereby, all SAx slices were used from the anulus of the atrioventricular valves to the apex at end-systole.

Septal flash volume was calculated accordingly as the difference of uncorrected LVESV and corrected ESV (Fig. 1b). Intra- and interobserver variability of the septal flash volume measurement were assessed in a subgroup 20 and 18 randomly selected individuals. For intraobserver variability, the same investigator acquired the measurements twice. For interobserver variability, two different and blinded investigators assessed the measurements separately.

The corrected LVESV was used to calculate the corrected LVEF. Corrected LVEF values of idiopathic LBBB patients were compared to LVEF reference values from our center (Additional file 1: Table S1). Two subgroups were identified; idiopathic LBBB patients with a normal LVEF and with a reduced LVEF after correction.

### Statistical analysis

Statistical analysis was performed using SPSS (version 24.0, Statistical Package for the Social Sciences, International Business Machines, Inc., Armonk, New York, USA) and MedCalc (version 15.7, MedCalc Software,Mariakerke, Belgium), with *p* < 0.05 taken as statistical significance for all tests. Continuous and normal distributed variables (Kolmogorov–Smirnov test, *p* ≥ 0.05) were expressed as mean ± standard deviation (SD). Group differences for continuous variables were tested using independent t-test or 2-way ANOVA for more than two groups followed up by Bonferroni post-hoc pairwise group comparisons. Continuous variables without normal distribution were stated as median and interquartile range, group differences were tested using nonparametric Mann–Whitney U test or Kruskal–Wallis test for more than two groups followed up by Bonferroni post-hoc pairwise group comparisons. Categorical variables were compared using chi-squared test. Pearson correlation was used to assess linear relationships between variables.

## Results

### Baseline characteristics

Baseline characteristics of patients and controls are presented in Table 1. All subjects completed CMR examination without complications. Fifty-three predominantly female patients (37 women, 70%) with a mean age of 60 ± 11 years (range from 35–79) constitute the idiopathic LBBB group. Patients in control group A were matched according to age, sex, BSA and CVD risk factors (Table 1). Patients in control group B were matched according to age, sex, BSA without any CVD risk factorsF (Table 1). QRS duration in the idiopathic LBBB group ranged from 120–170 ms (mean = 139 ± 13 ms) and septal flash volume was 10.2 ± 3.5 ml (indexed = 5.4 ± 1.7 ml/m^2^). Intraobserver variability of the septal flash volume was 0.96 (CI: 0.91–0.99) and interobserver variability 0.93 (CI: 0.81–0.97). NT-proBNP and high-sensitive Troponin T were within normal range in the idiopathic LBBB group.

**Table 1:** Baseline characteristics of patients with idiopathic left bundle-branch block (LBBB) and both age-, gender, and body mass index (BMI)-matched control groups with (A) and without (B) cardiovascular disease risk factors

Baseline characteristics	Idiopathic LBBB (n = 53)	Control group A CVRF ( +) (n = 53)	Control group B CVRF (−) (n = 26)	*p*	*p*
				(LBBB vs. control group A)	(LBBB vs. control group B)
Age (years)	60 ± 11	59 ± 10	59 ± 13	0.774	0.828
Female (n)	37 (70%)	37 (70%)	18 (69%)	1.000	0.958
Body surface area (m^2^)	1.86 (1.66–2.16)	1.87 (1.70–2.07)	1.87 (1.73–2.00)	0.950	0.867
Body mass index (kg/m^2^)	26 (23–31)	26 (23–30)	26 (24–27)	0.609	0.639
Heart rate (bpm)	71 ± 12	71 ± 14	68 ± 10	0.994	0.225
Hypertension	25 (48%)	13 (30%)	0 (0%)	0.064	< 0.001
Hypercholesterolemia	17 (34%)	7 (18%)	0 (0%)	0.090	< 0.05
Diabetes mellitus	6 (12%)	3 (7%)	0 (0%)	0.358	0.083
Smoking history	13 (26%)	10 (25%)	0 (0%)	0.914	< 0.01
Family history of coronary artery disease	12 (24%)	9 (21%)	0 (0%)	0.770	< 0.05
NT-proBNP (< 125 ng/l)	109 (83–218)				
High-sensitive Troponin T (< 14 pg/ml)	8 (5.8–12.3)				

*LBBB* left bundle-branch block; *bpm* beats per minute; *NT-proBNP* N-terminal pro B-type natriuretic peptide. Values are mean ± SD, median (IQR) or n (%)

Three patients revealed a mild stenosis on coronary angiography, but stress CMR examination was negative for ischemia. The median time between coronary angiography and CMR was 1 (0–13.5) month. Five patients underwent CMR more than 12 months after exclusion of CAD using coronary angiography, however stress CMR examination revealed no myocardial ischemia.

### Functional and structural remodeling in patients with idiopathic LBBB

Patients with idiopathic LBBB showed functional impairment and cardiac remodeling compared to controls:

1. LVEF, septal mitral annular plane systolic excursion (sMAPSE), GCS and GLS were significantly reduced in idiopathic LBBB compared to control groups A and B (LVEF, GCS, GLS: *p* < 0.001; sMAPSE: *p* < 0.01; Table 2).
2. LV end-diastolic volume (LVEDV) and indexed LVEDV (LVEDVI) were significantly enlarged in idiopathic LBBB patients compared to control group A and B (p < 0.001, Table 2), revealing a ventricular enlargement in the study group.
3. Global native T1 time was significantly increased in idiopathic LBBB patients compared to control group A and B at 3 T CMR (p < 0.05; Table 2, Additional file 1: Table S2). Also, septal native T1 times were significantly increased in idiopathic LBBB patients compared to control groups (p < 0.01; Table 2). However, there were no significant differences in lateral native T1 times between idiopathic LBBB patients and the control groups (Table 2). Global, septal and lateral native T1 times were significantly increased in the LBBB group compared to control group B (without CVRF) at the 1.5-T. Also, global T1 time at 3 T was significantly increased in LBBB patients compared to control group B (Table 2).

**Table 2:** CMR measures of left ventricular and right ventricular anatomy, function, native T1 and T2 in patients with idiopathic LBBB

CMR measurements	Idiopathic LBBB (n = 53)	Control group A CVRF ( +) (n = 53)	Control group B CVRF (−) (n = 26)	*p* (LBBB vs. control group A)	*p* (LBBB vs. control group B)
LVEF (%)	53.7 ± 4.6	62.9 ± 4.2	64.4 ± 5.0	< 0.001	< 0.001
sMAPSE (mm)	11 (10–12.5)	13 (11–14)	13 (11–16)	< 0.01	< 0.001
GCS	− 17.5 ± 3.0	− 20.5 ± 2.5	− 21.5 ± 2.2	< 0.001	< 0.0001
GLS	− 15.7 ± 3.0	− 18.2 ± 2.3	− 19.7 ± 3.2	< 0.001	< 0.0001
LVEDV (ml)	156.6 ± 39.3	135.5 ± 24.8	133.3 ± 28.0	< 0.01	< 0.01
LVEDVI (ml/m^2^)	82.5 ± 14.9	71.8 ± 9.9	71.4 ± 13.1	< 0.001	< 0.01
RVEDV (ml)	129.9 ± 30.5	130.3 ± 31.4	141.3 ± 33.9	0.940	0.228
RVEDVI (ml/m^2^)	68.4 ± 11.3	68.8 ± 11.8	73.3 ± 13.9	0.871	0.181
RVEF (%)	64.4 ± 6.6	65.0 ± 6.7	61.8 ± 6.3	0.641	0.191
TAPSE (mm)	21 (19–24)	22 (19.5–26)	22 (19–25)	0.120	0.346
LV mass (g)	97 ± 27	88 ± 22	87 ± 20	0.077	0.104
Native T1					
1.5 T	n = 22	n = 21	n = 13		
Global (ms)	1023 ± 25	1009 ± 20	1000 ± 20	0.060	< 0.05
Septal (ms)	1046 ± 30	1022 ± 22	1006 ± 17	< 0.01	< 0.001
Lateral (ms)	1017 ± 27	1006 ± 29	988 ± 28	0.185	< 0.01
3 T	n = 19	n = 26	n = 13		
Global (ms)	1260 ± 39	1238 ± 25	1216 ± 64	< 0.05	< 0.05
Septal (ms)	1293 ± 56	1256 ± 30	1252 ± 26	< 0.01	0.062
Lateral (ms)	1225 ± 42	1226 ± 30	1219 ± 37	0.981	0.723
T2					
1.5 T	n = 12	n = 19	n = 12		
Global (ms)	53.5 ± 2.3	53.8 ± 1.8	52.1 ± 1.8	0.094	0.709
3 T	n = 6	n = 24	n = 11		
Global (ms)	45.9 ± 3.0	47.7 ± 2.3	46.5 ± 2.6	0.137	0.698

*CVRF* cardiovascular risk factors, *LBBB* left bundle-branch block, *LV* left ventricle, *EDV* end-diastolic volume, *EDVI* end-diastolic volume index, *EF* ejection fraction, *sMAPSE* septal mitral annular plane systolic excursion, *GCS* global circumferential strain, *GLS* global longitudinal strain, *RV* right ventricle, *TAPSE* tricuspid annular plane systolic excursion; Comparison of native T1 and T2 between LBBB patients and controls in 1.5 T and 3 T: Values are mean ± SD or median (interquartile range)

There were no significant differences in global T2 times between idiopathic LBBB patients and control group A and B. (Table 2). Additionally, there were no abnormal segmental T2 times (center-specific cut-off 58 ms). Therefore, a relevant myocardial edema could not be detected and elevated global and septal T1 times indicate myocardial fibrosis.

4. Results from LGE highlight that myocardial fibrosis occurs in patients with idiopathic LBBB. LGE was performed in 40 LBBB patients. Seven patients revealed non-ischemic LGE at the inferior right ventricular insertion point and one patient at the anterior right ventricular insertion point, while none of the patients in control group A or B showed LGE. LV enlargement, increased global and septal T1 times and LGE at the inferior right ventricular insertion point demonstrate structural remodeling in idiopathic LBBB patients.

5. There were no significant differences in normal and indexed right ventricle (RV) EDV (RVEDVI), RVEF, tricuspid annular plane systolic excursion (TAPSE) and LV mass between the idiopathic LBBB and control group A or B (Table 2, Additional file 1: Table S2). However, there was a negative correlation between RVEF (r = -0.275, r^2^ = 0.076, *p* < 0.05) and a positive correlation between RVEDVI and QRS duration (r = 0.599, r^2^ = 0.359, *p* < 0.05) (Table 3, Additional file 1: Table S3) indicating functional and structural remodeling also at the RV in idiopathic LBBB patients.

**Table 3:** Correlation coefficients between QRS duration and functional and structural measurements in patients with idiopathic LBBB

QRS duration (ms)	r^2^	r	p
LVEF (%)	0.449	− 0.670	< 0.001
Flash volume indexed (ml/m^2^)	0.304	0.551	< 0.001
LVEDVI (ml/m^2^)	0.461	0.679	< 0.001
LV mass (g)	0.527	0.726	< 0.001
RVEF (%)	0.076	− 0.275	< 0.05

* LBBB* left bundle-branch block, *LV* left ventricle, *EDV* end-diastolic volume, *EDVI* end-diastolic volume index, *EF* ejection fraction, *RV* right ventricle. *r*^2^ coefficient of determination. Pearson correlation was used to calculate linear relationships between CMR and QRS duration

### Correlation of functional impairment, structural remodeling and QRS duration

**Fig. 2:**
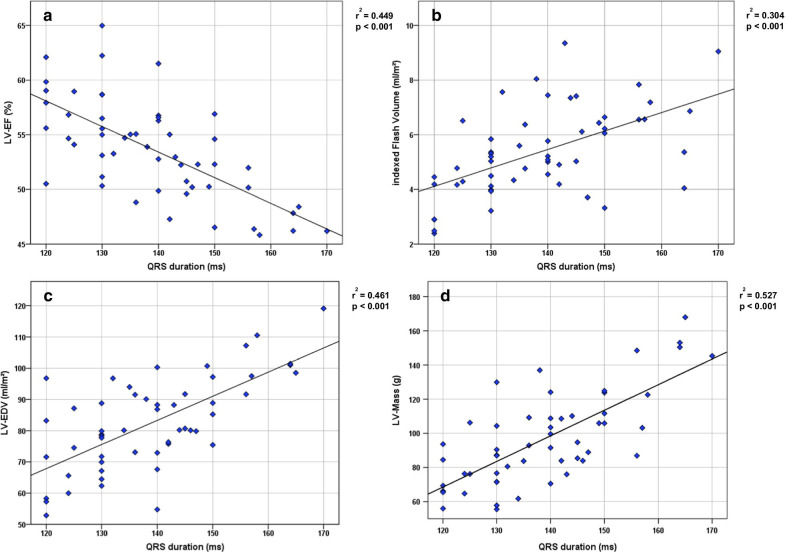
Correlation of structural remodeling, functional impairment and QRS duration. Correlation between left ventricular (LV) ejection fraction (LVEF) (**a**), indexed Flash Volume (**b**), indexed left ventricular end-diastolic volume (LVEDVI) (**c**) and LV end-systolic volume (LVESV) (**d**) to QRS duration. Pearson correlation was used, r^2^- and p-values as stated

LVEF was inversely correlated (r = − 0.670, r^2^ = 0.449,* p* < 0.001, Fig. 2c, Table 3), while indexed septal flash volume was positively correlated with QRS duration (r = 0.551, r^2^ = 0.304, *p *< 0.001, Fig. 2d, Table 3). Therefore, functional impairment in idiopathic LBBB patients correlates with the degree of ventricular conduction delay. LVEDVI and LV mass was positively correlated with QRS duration (p < 0.001; Fig. 2a, Table 3).

### Superimposed functional and structural impairment by idiopathic LBBB

**Fig. 3:**
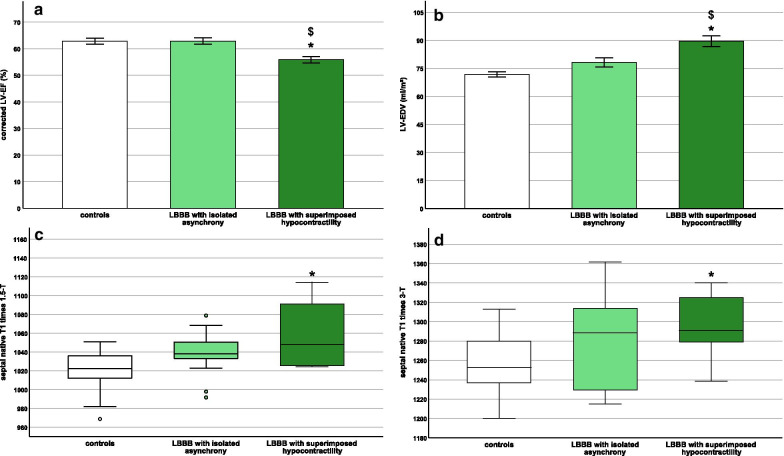
Superimposed contractile impairment in patients with idiopathic LBBB. Comparison of idiopathic LBBB patients with isolated ventricular asynchrony (LBBB with isolated asynchrony), with superimposed contractile impairment (LBBB with superimposed hypocontractility) and controls: corrected left ventricular ejection fraction (corrected LVEF) (**a**), left ventricular end-diastolic volume (LVEDV) (**b**), septal native T1 times in 1.5 T (**c**) and in 3 T (**d**). Differences between all three groups were calculated using 2-way ANOVA, Bonferroni-Posttest between all three subgroups, *p < 0.05 vs. controls, ^$^*p* < 0.05 vs. LBBB with isolated asynchrony

Thirty-three patients with idiopathic LBBB were identified having a normal LVEF after correction of septal flash volume, revealing systolic dysfunction solely due to isolated ventricular asynchrony (LBBB with isolated asynchrony). However, 22 patients had a reduced LVEF after correction of septal flash volume, indicating a superimposed contractile impairment (LBBB with superimposed hypocontractility). Indexed septal flash volume was similar in both groups (LBBB with isolated asynchrony = 5.3 ± 1.5 ml/m^2^; LBBB with superimposed hypocontractility = 5.4 ± 1.7 ml/m^2^; *p* = 0.720). The time between first documented LBBB in ECG and the CMR examination was similar between both groups (LBBB with isolated asynchrony = 83 (18–112) days; LBBB with superimposed hypocontractility = 78 (47–617) days; p = 0.777). However, significant differences between the groups were found:

1. LVEF, GCS and GLS were significantly lower in the group with a superimposed hypocontractility compared to the fraction with isolated ventricular asynchrony (*p* < 0.001, *p* < 0.01 and *p* < 0.05 respectively, Fig. 3a, Table 4). GCS and GLS were significantly reduced in both subgroups compared to the control groups (Tables 4 and 5). There were no significant differences in NT-proBNP and high-sensitive Troponin T between the group with a superimposed hypocontractility compared to the group with isolated asynchrony. However, NT-proBNP was slightly above the 99th percentile in LBBB patients with a superimposed hypocontractility (Table 4).

**Table 4:** Comparison of idiopathic LBBB patients with isolated ventricular asynchrony, with superimposed contractile impairment and control group A with cardiovascular  disease risk factors

CMR measurements	LBBB with isolated asynchrony (n = 33)	LBBB with superimposed hypocontractility (n = 20)	control group A CVRF ( +) (n = 53)	ANOVA	Bonferroni Post-Hoc	LBBB with isolated asynchrony vs. control group A	LBBB with superimposed hypocontractility vs. control group A
				*p*	LBBB with isolated asynchrony vs. LBBB with superimposed hypocontractility		
LVEDVI (ml/m^2^)	78.1 ± 14.3	89.8 ± 14.0	71.8 ± 9.9	< 0.001	< 0.01	0.059	< 0.001
corrected LV-EF (%)	62.9 ± 3.6	55.9 ± 2.8	62.9 ± 4.2	< 0.001	< 0.001	1.000	< 0.001
RV-EDVI (ml/m^2^)	65.0 ± 9.7	74.2 ± 11.7	68.8 ± 11.8	< 0.05	< 0.05	0.382	0.209
RV-EF (%)	66.9 ± 5.3	60.4 ± 6.8	65.0 ± 6.7	< 0.05	< 0.01	0.576	< 0.05
GCS	− 18.5 ± 2.9	− 15.9 ± 2.4	− 20.5 ± 2.5	< 0.001	< 0.01	< 0.01	< 0.001
GLS	− 16.4 ± 3.3	− 14.5 ± 1.8	− 18.2 ± 2.3	< 0.001	< 0.05	< 0.05	< 0.001
LV mass (g)	93 ± 25	103 ± 30	88 ± 22	0.068	0.397	1.000	0.062
Native T1 times							
1.5 T	n = 14	n = 8	n = 22				
Global (ms)	1015 ± 24	1035 ± 24	1009 ± 20	< 0.05	0.144	1.000	< 0.05
Septal (ms)	1039 ± 24	1059 ± 36	1022 ± 22	< 0.01	0.271	0.185	< 0.01
Lateral (ms)	1015 ± 30	1021 ± 23	1006 ± 29	0.377	1.000	1.000	0.606
3 T	n = 11	n = 8	n = 26				
Global (ms)	1253 ± 35	1271 ± 43	1238 ± 25	< 0.05	0.651	0.575	< 0.05
Septal (ms)	1282 ± 54	1308 ± 60	1256 ± 30	< 0.05	0.578	0.294	< 0.05
Lateral (ms)	1222 ± 28	1230 ± 57	1226 ± 30	0.875	1.000	1.000	1.000
LGE	n = 3	n = 5		T-Test			
				p			
Enhanced myocardial mass (g)	1.01 ± 0.11	1.99 ± 0.43	–	< 0.01	–	–	–
Relative enhanced myocardial mass (%)	1.27 ± 0.26	2.00 ± 0.44	–	< 0.05	–	–	–
Biomarkers				Mann–Whitney-Test			
NT-proBNP (< 125 ng/l)	99 (49–152)	133 (104–239)	–	0.231	–	–	–
High sensitive Troponin T (< 14 pg/ml)	11 (7–16)	6 (5–11)	–	0.063	–	–	–

*CVRF* cardiovascular risk factors, *LV* left ventricle, *EDVI* end-diastolic volume index, *EF* ejection fraction, *RV* right ventricle, *GCS* global circumferential strain, *GLS* global longitudinal strain, *LGE* late gadolinium enhancement, *NT-proBNP* N-terminal pro B-type Natriuretic Peptide. Values are mean ± standard deviation. Differences between all three groups were calculated using 2-way ANOVA and Bonferroni-Posttest. Differences in between the LBBB subgroups were calculated using T-Test or Mann–Whitney-Test

**Table 5:** Comparison of idiopathic LBBB patients with isolated ventricular asynchrony, with superimposed contractile impairment and control group B without cardiovascular risk factors

CMR measurements	LBBB with isolated asynchrony (n = 33)	LBBB with superimposed hypocontractility (n = 20)	Control group B CVRF (−) (n = 26)	ANOVA p	Bonferroni Post-Hoc	LBBB with isolated asynchrony vs. control group B	LBBB with superimposed hypocontractility vs. control group B
					LBBB with isolated asynchrony vs. LBBB with superimposed hypocontractility		
LVEDVI (ml/m^2^)	78.1 ± 14.3	89.8 ± 14.0	71.4 ± 13.1	< 0.001	< 0.01	0.013	< 0.0001
corrected LV-EF (%)	62.9 ± 3.6	55.9 ± 2.8	64.4 ± 5.0	< 0.001	< 0.001	0.450	< 0.001
RVEDVI (ml/m^2^)	65.0 ± 9.7	74.2 ± 11.7	73.3 ± 13.9	< 0.05	< 0.05	0.072	1.000
RV-EF (%)	66.9 ± 5.3	60.4 ± 6.8	61.8 ± 6.3	< 0.05	< 0.01	0.294	1.000
GCS	− 18.5 ± 2.9	− 15.9 ± 2.4	− 21.5 ± 2.2	< 0.001	< 0.01	< 0.001	< 0.001
GLS	− 16.4 ± 3.3	− 14.5 ± 1.8	− 19.7 ± 3.2	< 0.001	< 0.05	< 0.001	< 0.001
LV mass (g)	93 ± 25	103 ± 30	87 ± 20	0.090	0.420	1.000	0.088
Native T1 times							
1.5-T	n = 14	n = 8	n = 13				
Global (ms)	1015 ± 24	1035 ± 24	1000 ± 20	< 0.01	0.144	0.303	< 0.05
Septal (ms)	1039 ± 24	1059 ± 36	1006 ± 17	< 0.001	0.271	< 0.05	< 0.001
Lateral (ms)	1015 ± 30	1021 ± 23	988 ± 28	< 0.05	1.000	0.069	0.050
3-T	n = 11	n = 8	n = 13				
Global (ms)	1253 ± 35	1271 ± 43	1216 ± 64	0.056	0.651	0.275	0.070
Septal (ms)	1282 ± 54	1308 ± 60	1252 ± 26	0.096	0.578	0.614	0.097
Lateral (ms)	1222 ± 28	1230 ± 57	1219 ± 37	0.850	1.000	1.000	1.000

*LBBB* left bundle-branch block, *CVRF* cardiovascular risk factors, *LV* left ventricle, *EDV* end-diastolic volume, *EDVI* end-diastolic volume undex, *EF* ejection fraction, *RV* right ventricle; *GCS* global circumferential strain, *GLS* global longitudinal strain. Values are mean ± standard deviation. Differences between all three groups were calculated using 2-way ANOVA and Bonferroni-Posttest

**Fig. 4:**
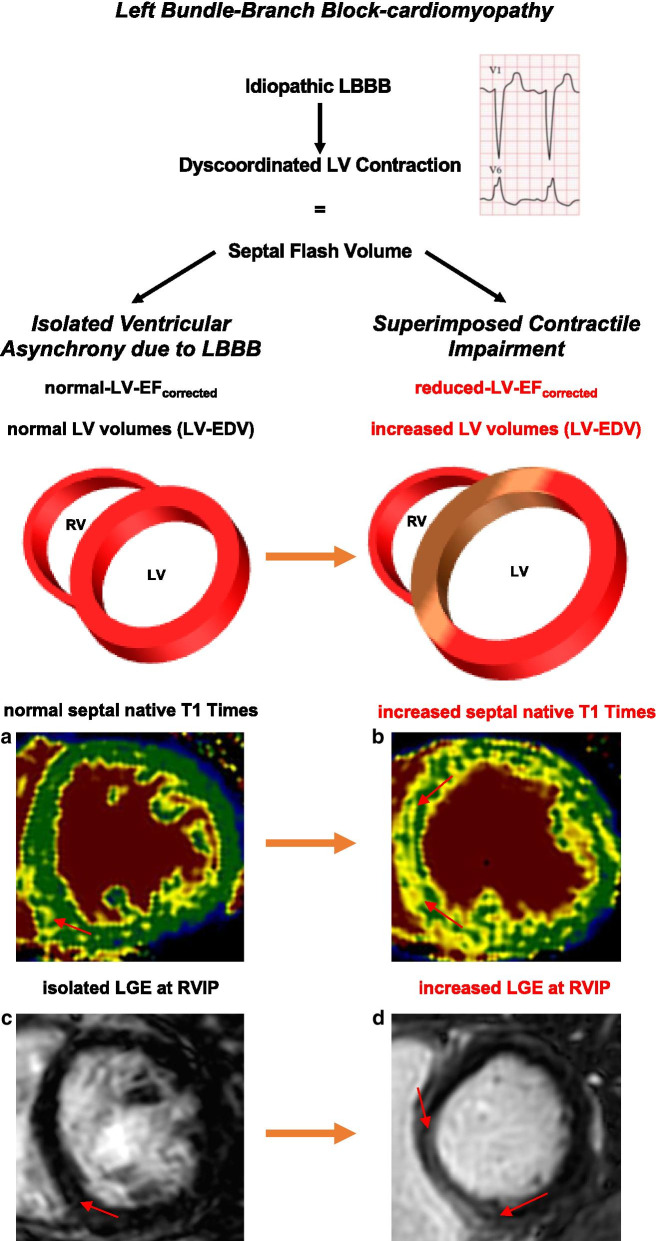
Left Bundle-Branch Block-cardiomyopathy. The figure represents two subgroups of LBBB-cardiomyopathy; Patients with isolated ventricular asynchrony and patients with superimposed contractile impairment. **a**, **b** representative images of native T1 in patients with isolated ventricular asynchrony (**a**) and patients with superimposed contractile impairment (**b**). Colors(1.5 T); green: 955-1052 ms; yellow: 1053-1150 ms; brown 1151-1400 ms. **c**, **d** representative images of late gadolinium enhancement (LGE) at the inferior insertion point of the right ventricular free wall (RVIP) of patients with isolated ventricular asynchrony (**c**) and patients with superimposed contractile impairment (**d**). Orange arrows indicate a possible development of a superimposed contractile impairment in patients with idiopathic LBBB. *LV* left ventricle, *EF* ejection fraction EDV-end-diastolic volume, *RV* right ventricle

2. LVEDVI was significantly increased in the group with a superimposed hypocontractility compared to the one with isolated asynchrony (*p* < 0.001, Fig. 3b, Table 4). Interestingly, LVEDVI did not significantly differ between the LBBB group with isolated asynchrony and the control groups (Fig. 3b, Table 4). This indicates that structural remodeling with LV enlargement only occurs in a subgroup of idiopathic LBBB patients with superimposed contractile impairment.

3. Global and septal native T1 times were significantly increased in the group with superimposed contractile impairment compared to both control groups (*p* < 0.05, Fig. 3c, d, Tables 4, 5, Additional file 1: Table S4). However, there were no significant differences in global or septal native T1 times between the idiopathic LBBB group with isolated ventricular asynchrony and the controls.

4. Additionally, LGE was significantly more present in the group with superimposed hypocontractility compared to the one with isolated asynchrony (enhanced myocardial mass (g): *p* < 0.01; relative enhanced LV mass (%): *p* < 0.05, Table 4). The increased global and septal T1 times and the LGE indicate that myocardial fibrosis primarily occurs in the subgroup with a superimposed contractile impairment (Fig. 4).

5. RVEDVI was significantly increased and RVEF decreased in the subgroup with superimposed contractile impairment compared to one with isolated ventricular asynchrony (*p* < 0.05, Table 4). This reveals that functional impairment and structural remodeling also occurs in the RV in a subgroup of idiopathic LBBB patients.

## Discussion

This study suggests two separate mechanisms in patients with idiopathic LBBB that are both contributing in a differing extent to systolic impairment and structural remodeling. To our knowledge no data are available in the literature demonstrating this mechanism. First, the isolated ventricular asynchrony results in functional impairment, because septal flash volume, as the volume displaced from the lateral wall to the septum, does not contribute to the global stroke volume resulting in a reduced ejection fraction. Hence, LV dysfunction can be attributed solely to the effect of septal flash volume in a portion of patients. However, in close to a half of the studied individuals septal flash volume alone did not explain the apparent systolic impairment sufficiently. In this fraction of patients with idiopathic LBBB we assume an additional superimposed contractile impairment and could demonstrate an increased structural remodeling compared to those with isolated ventricular asynchrony (Fig. 4). These patients showed increased septal native T1 times compared to patients with septal flash volume effects alone and to controls. Additionally, LGE was significantly more present in LBBB patients with a superimposed contractile impairment compared to patients with isolated ventricular asynchrony, even though the relative difference was just above 35%. These findings are very suggestive for an increased amount of histologically present interstitial fibrosis. Previous studies demonstrated a correlation between T1 with extracellular volume fraction [17]. Differences in T1 mapping were found between healthy individuals and patients with cardiomyopathies, such as dilated cardiomyopathy [18]. There was no clear indication of myocardial edema in LBBB patients as global and regional myocardial T2 times were normal, even though T2 measurements were not available in all patients. Hence, the increased native T1 times in particular in the group with superimposed contractile impairment suggests myocardial fibrosis. Additionally, non-ischemic LGE at the inferior right ventricular insertion point confirms myocardial fibrosis primarily in this group, even though LGE was not available in all patients with idiopathic LBBB. Myocardial fibrosis indicates increased cardiac remodeling and goes along with pathologic myocardial stiffening and reduced ventricular function as previously demonstrated [19]. Also, patients with superimposed contractile impairment showed slightly elevated NT-proBNP levels (above the 99^th^ percentile), indicating increased chronic myocardial damage.

These findings demonstrate the pathophysiologic heterogeneity of idiopathic LBBB. The evidence for a superimposed hypocontractility in addition to effects solely due to septal flash volume and therefore isolated ventricular asynchrony suggests a LBBB-associated cardiomyopathy (Fig. 4). However, future studies will have to address the eminent question, if a classification based on these characteristics might serve as a relevant determinant of individual prognosis. Regarding this aspect, previous studies of non-ischemic cardiomyopathy could demonstrate a worse prognosis in patients with LGE [20]. Additionally, GCS and GLS are powerful independent predictors for cardiac events in patients with non-ischemic cardiomyopathies [21, 22]. Therefore, reduced GCS and GLS in patients with isolated LBBB and in particular in the group with superimposed hypocontractility might indicate a higher risk for the occurrence of cardiac events such as sudden cardiac death, ventricular arrhythmias and hospitalization due to heart failure. Larsen et al. revealed a reduced myocardial strain in particular in the septal segments, which stands in line with our results [23].

In addition to these findings, we demonstrated that CMR does allow a quantification of septal flash volume with a high intra- and interobserver variability, which was previously only assessed visually by blinded physicians in echocardiographic studies [24, 25]. Septal flash volume was positively correlated with QRS duration, revealing a clear correlation between cardiac dyssynchrony and the degree of conduction delay caused by LBBB, indicating an electro-mechanical dissociation. In our study patients with idiopathic LBBB suffered from a reduced LVEF and increased LVEDV compared to age-, sex-, BSA- and CVD risk factor-matched individuals and compared to healthy individuals without CVD risk factors, which confirms findings from previous echocardiography studies [25, 26]. Also similar to previous published studies using CMR, which is currently considered as the gold standard for the evaluation of LV function and volumes, increased LVEDV and reduced LVEF in patients with idiopathic LBBB compared to healthy controls was shown [1, 27]. Additionally, our data revealed a correlation between functional impairment and structural remodeling and the degree of ventricular conduction delay in patients with idiopathic LBBB by showing that LVEF was inversely correlated and LVEDV was positively correlated with QRS duration. Valenti et al. measured interventricular dyssynchrony in patients with idiopathic LBBB, which was defined as the temporal difference between the onset of aortic and pulmonary flow and was positively correlated with LVEDVI [27], which stands in line with our results. RVEDVI and ESV were correlated with QRS duration, while RVEF was inversely correlated with QRS duration, indicating that functional and structural alterations also occur in the RV.

### Clinical context

Exercise intolerant patients with idiopathic LBBB and systolic impairment caused by septal flash volume effects alone might benefit more extensively from CRT than patients with a leading cause of reduced global contractile function. This hypothesis is supported by Vaillant et al., who demonstrated the development of heart failure in idiopathic LBBB patients and the recovering of LVEF after CRT implantation [7]. Even though the retrospective study had a small sample size, it demonstrates that LBBB-cardiomyopathy reduces cardiac function, but seems to be a potentially reversible process. In contrast, patients suffering from LBBB-cardiomyopathy with superimposed contractile impairment and fibrosis might have an irreversible structural remodeling and hence do not respond satisfactorily to CRT. CRT is a well-established treatment in patients with heart failure with reduced LVEF [28]. However, about 30% of patients do not respond to CRT [29]. Differentiating patients with cardiac dysfunction solitary due to ventricular asynchrony caused by LBBB from those with a superimposed LBBB-cardiomyopathy might help to reduce the rate of CRT non-responders.

### Limitations

The present study has some limitations, such as the retrospective design. It is possible that patients with isolated ventricular dyssynchrony develop LBBB-cardiomyopathy with fibrosis and superimposed hypocontractility over time. However, in order to proof a sequential cascade of this pathology, knowledge about the onset and duration of LBBB is necessary. The onset of LBBB was similar in both groups, indicating that the development of a superimposed hypocontractility is independent of the duration of LBBB. However, due to the retrospective nature of this study, the onset point of LBBB was not available in all patients and the large interquartile range in the LBBB group with superimposed hypocontractility indicates a heterogeneity in this group. Therefore, longitudinal prospective follow-up studies are required to investigate the development of myocardial fibrosis in patients with idiopathic LBBB. Also, prospective follow-up studies would be necessary to evaluate if patients may develop clinically relevant heart failure with reduced ejection fraction or a cardiomyopathy.

Additionally, due to incomplete data of native T1 maps, LGE and T2 measurements, the degree of fibrosis could not be investigated in all patients. Only about 2% of patients with a documented LBBB or dyscoordinated contraction had an isolated LBBB, indicating that it is difficult even in a tertiary referral hospital to identify suitable individuals. However, the present study consists, to our knowledge, of the largest population with idiopathic LBBB studied using CMR.

Coronary angiography was not performed in all patients in order to exclude CAD. Even though the rest of the cohort had a negative stress CMR or echo and none of the LBBB patients had cine CMR evidence for regional dysfunction other than the septum, invasive coronary angiography or coronary computed tomography angiography should be performed to rule out CAD in future studies. The high prevalence of female patients (70%) is most likely a result of the retrospective nature of the study. Our study consists of a relatively small sample size. However, it needs to be emphasized that among 2356 patients with a documented LBBB, only 53 fulfilled the criteria for our study. Therefore, additional prospective studies are required in order to exclude a sex-specific functional impairment in patients with idiopathic LBBB. Additionally,  the measurements of T1 values with three different vendors including a 1.5 T and 3 T scanner resulted in different T1 times. This limits the number of individuals that can be compared between the patient group and the control group as well as between the subgroups. In future studies, the same vendor should be used for the study and the control group. Also, extracellular volume fraction (ECV) would be useful for quantification of myocardial fibrosis in further detail. Furthermore, to our knowledge this is the first study that measured septal flash volume using CMR. Additional studies are necessary to confirm that septal flash volume influences cardiac dysfunction and structural remodeling using CMR. Patients with idiopathic LBBB and controls had similar prevalence of hypertension, however we do not know the exact blood pressure during CMR examination. Moderate elevation of blood pressure can reduce LVEF and GLS in patients with LBBB [30]. Therefore, blood pressure should be monitored before CMR is performed to exclude this potential confounder. Although cardiac biomarkers (NT-pro BNP and Troponin T) were available, in some cases blood samples were drawn a significant time before the CMR examination. Therefore, elevated NT-pro BNP may not represent chronic myocardial damage.

In addition, genetic data are missing in this study, which might help to distinguish global hypocontractility and fibrosis caused by LBBB from other underlying cardiomyopathies such as dilated cardiomyopathy. While our data demonstrate that idiopathic LBBB induces cardiac dysfunction and structural remodeling, prospective studies are required to investigate potential risk factors, time of onset and development of myocardial fibrosis and finally prognosis of these patients.

## Conclusions

There is heterogeneity of cardiac remodeling in patients with idiopathic LBBB suggesting two separate pathophysiologic mechanisms that are both contributing in a differing extent to systolic impairment and structural remodeling. Besides isolated ventricular asynchrony, patients with hypocontractility have been identified indicative for the presence of an LBBB-associated cardiomyopathy.

## Supplementary Information

**Additional file 1.** Additional tables.

## Data Availability

The datasets used and analysed during the current study are available from the corresponding author on reasonable request.

## Abbreviations

BMI: Body mass index
BSA: Body-surface area
bSSFP: Balanced steady state free precession
CAD: Coronary artery disease
CMR: Cardiovascular magnetic resonance
CRT: Cardiac resynchronization therapy
CVD: Cardiovascular disease
ECG: Electrocardiogram
ECV: Extracellular volume fraction
EDV: End-diastolic volume
EDVI: End-diastolic volume index
EF: Ejection fraction
ESV: End-systolic volume
GCS: Global circumferential strain
GLS: Global longitudinal strain
LA: Left atrium
LBBB: Left bundle-branch block
LGE: Late gadolinium enhancement
LV: Left ventricle/left ventricular
LVEDV: Left ventricular end-diastolic volume
LVEDVI: Left ventricular end-diastolic volume index
LVEF: Left ventricular ejection fraction
LVESV: Left ventricular end-systolic volume
MOLLI: Modified look-locker inversion recovery
RA: Right atrium
RV: Right ventricle/right ventricular
RVEDV: Right ventricular end-diastolic volume
RVEDVI: Right ventricular end-diastolic volume index
RVEF: Right ventricular ejection fraction
SAx: Short-axis
sMAPSE: Septal mitral annular plane systolic excursion
SV: Stroke volume
TAPSE: Tricuspid annular planse systolic excursion
