# Supplementary material for: Presence of contractile impairment appears crucial for structural remodeling in idiopathic left bundle-branch block
Source: J Cardiovasc Magn Reson. 2021 Apr 1;23:39. doi: 10.1186/s12968-021-00731-6 (PMC8015193; doi:10.1186/s12968-021-00731-6)
Supplement: Supplementary file 1 — Additional file 1. Additional tables. [file 12968_2021_731_MOESM1_ESM.docx]

**Supplementary material**

**Title: Presence of contractile impairment appears crucial for structural remodeling in idiopathic Left Bundle-Branch Block.**

**Names of authors**

Janek Salatzki, MD,^a,b^ Theresa Fischer,^a^ Johannes Riffel, MD,^a,b^ Florian André, MD,^a,b^ Kristóf Hirschberg, MD, PHD,^a,c^ Andreas Ochs, MD,^a,b^ Hauke Hund,^a^ Matthias Müller-Hennessen, MD,^a,b^ Evangelos Giannitsis, MD,^a,b^ Matthias G. Friedrich, MD,^a,b,d^ Eberhard Scholz, MD,^a,b^ Norbert Frey, MD,^a,b^ Hugo A. Katus, MD,^a,b^ Marco Ochs, MD,^a,b^

**Institutions**

^a^Department of Cardiology, Angiology and Pneumology, Heidelberg University Hospital, Heidelberg, Germany

^b^Semmelweis University Heart and Vascular Center, Budapest, Hungary

^c^Division of Cardiology, Departments of Medicine and Diagnostic Radiology, Mc-Gill University Health Centre, Montreal, Canada

**Methods**

**Cardiovascular magnetic resonance image acquisition protocol and post-processing**

Following localizing scans, cine long axis 2-, 3- and 4-chamber views as well as short axis cine images covering the whole LV from the anulus of the atrioventricular valves to the apex (8 mm slice thickness, no gap between each slice) were obtained using a breath-hold, segmented-k-space balanced steady-state free precession sequence (bSSFP) employing retrospective ECG or pulse oximetric gating with 40 (1.5-T Achieva) or 35 (1.5-T and 3-T) phases per cardiac cycles. Scan parameters were: repetition time (TR) 2.8ms; echo time (TE) 1.4ms; flip angle (FA) 60° (1.5-T Achieva and 1.5-T), TR 2.9ms, TE 1.4ms, FA 45° (3T); with a breath-hold time of 7–10 s per image and prospective gating. Data were analyzed using the CVI cmr^42^ software (Version 5.6.6, Circle Cardiovascular Imaging Inc., Calgary, Canada) as semi-automatic software for volumetric analysis. Ventricular volumes, ejection fraction of LV and right ventricle (RV) and LV myocardial mass were acquired in short axis stacks by manually tracing epi- and endocardial borders, excluding papillary muscles from the myocardium.

**Myocardial tissue characterization**

In order to measure T1 times, Myocardial T2 times were measured using the following parameters: TR was one RR interval, 9 echoes, TE 18ms, FA 90°. After visual inspection of all segments and exclusion of those segments with evidence of artefact, a mean T1 and T2 relaxation time for the remaining segments of each slice was calculated. If more than two segments showed evidence of artefacts, the case was excluded from further analysis. Endocardial and epicardial borders were defined manually, using an offset of 10% to avoid partial-volume effects in the subendocardial and subepicardial layers using the CVI cmr^42^ software.

Regional T1 times are presented for anterior, antero-lateral, infero-lateral, inferior, infero-septal, antero-septal segments using T1 mapping. T1-times of the segments antero-septal and infero-septal were combined and labelled “septal”, while segments antero-lateral and infero-lateral were combined and labelled “lateral”. Global T1 and T2 was measured by calculating the mean over all segments.

Late gadolinium enhancement (LGE) images were acquired 10 minutes after administration of gadolinium diethylenetriamine pentaacetic acid /DTPA (Magnograf, Schering, Berlin, Germany) 0.2 mmol/ kg body weight (before February 2016) or 10 minutes after administration of Gadobutol (Gadovist, Schering, Berlin, Germany) 0.14 mmol/ kg body weight (1.5-T) or 0.1 mmol/ kg body weight (3-T) (after February 2016) employing a T1-weighted inversion recovery-prepared fast gradient echo sequence with an optimized inversion time. Regions with LGE were verified in at least one other orthogonal plane and in the same plane being obtained as a second image after changing the direction of readout. LGE was quantified using the CVI cmr^42^ software and defined as areas with a signal intensity more than 5 standard deviations higher than the mean signal intensity of remote myocardium in the same short-axis section (1).

**Results**

**Functional and Structural Remodeling in patients with idiopathic LBBB**

LV end-systolic volume (LV-ESV) and indexed LV ESV were significantly increased in patients with idiopathic LBBB compared to controls (p<0.001, Table S1). There were no significant differences in indexed LV stroke volume (LV-SV), LV cardiac output (LV-CO), LV mass and wall thickness (septum and lateral wall) between idiopathic LBBB patients and controls. There were no significant differences in (indexed) RV-ESV, RV-SV, RV-CO between the idiopathic LBBB and control group. There were also no significant differences in the size of the left (LA) and right atrium (RA) (Table S1).

Native T1 times of the infero-septal and antero-septal segments were significantly increased in idiopathic LBBB patients compared to controls (infero-septal; p<0.05; antero-septal; 1.5-T: p<0.05; 3-T: p<0.01, Table S1). Native T1 times in the anterior segment was significantly increased at 3-T (p<0.05, Table S1). There were no significant differences in native T1 times of the anterior-lateral, inferior-lateral and inferior segments between idiopathic LBBB patients and controls (Table S1).

**Correlation of functional impairment, structural remodeling and QRS duration**

Indexed LV-ESV was positively correlated with QRS duration with r=0.730, r^2^=0.533 (p<0.001). Indexed LV-CO did not correlate with QRS duration. Indexed RV-EDV and RV-ESV were positively correlated with QRS duration with r=0.599, r^2^=0.359 (p<0.001) and r=0.479, r^2^=0.229 (p<0.001) respectively. Indexed RV-CO did not correlate with QRS duration (Table S2).

**Superimposed functional and structural impairment by idiopathic LBBB**

Indexed LV-ESV was significantly increased in the group with superimposed contractile impairment and in the one with isolated ventricular asynchrony compared to controls (p<0.001, Table S3). There were no significant differences in indexed LV-CO, RV-CO and LV mass between patients with idiopathic LBBB and superimposed contractile impairment, isolated ventricular asynchrony and controls. Indexed RV-ESV was significantly increased in the group superimposed contractile impairment compared to the group with isolated ventricular asynchrony (p<0.001) and compared to controls (p<0.05) (Table S3). There were no significant differences in native T1 times of the infero-lateral and inferior segment between the LBBB group with isolated asynchrony, the LBBB group with superimposed hypocontractility and controls (Table S3). The anterior and antero-lateral segments of the LBBB group with superimposed hypocontractility showed significantly increased native T1 times compared to controls using the 3-T (p<0.05, Table S3). In addition, the infero-septal and antero-septal segment of the LBBB group with superimposed hypocontractility showed significantly increased native T1 times compared to controls (p<0.05, Table S3).

Table S1 Center-specific reference values for ejection fraction.

| **Normal range of EF(%) for center** | **Gender** | |
| --- | --- | --- |
| **Age (years)** | Female | Male |
| 20-40 | 58-72 | 53-73 |
| 40-60 | 60-75 | 56-76 |
| older than 60 | 61-78 | 59-79 |

Normal range of ejection fraction (EF) according to age groups (20-40 years, 40-60 years and older than 60 years) and gender.

Table S2 Cardiac Magnetic Resonance Measurements of left and right ventricle and native T1 times in patients with idiopathic LBBB.

| **CMR Measurements** | **idiopathic LBBB** (n=53) | **Controls** (n=53) | **p** |
| --- | --- | --- | --- |
| LV-ESV (ml) | 73.7 ± 24.1 | 50.6 ± 12.0 | <0.001 |
| LV-ESV indexed (ml/m²) | 38.7 ± 10.0 | 26.8 ± 5.5 | <0.001 |
| LV-SV (ml) | 82.9 ± 16.8 | 84.9 ± 15.1 | 0.513 |
| LV-SV indexed (ml/m²) | 43.7 ± 5.7 | 45.0 ± 5.9 | 0.272 |
| LV-CO (l/min) | 5.8 ± 1.3 | 6.0 ± 1.3 | 0.585 |
| LV-CO indexed (l/min/m²) | 3.1 ± 0.5 | 3.2 ± 0.7 | 0.389 |
| Septum (mm) | 10 (9-11) | 9 (8-10) | 0.102 |
| Lateral Wall (mm) | 6 (6-7.5) | 6 (5-7) | 0.156 |
| LA Diameter (mm) | 35 (32-38) | 34 (30-38) | 0.200 |
| RV-ESV (ml) | 47.0 ± 16.8 | 47.0 ± 18.8 | 0.995 |
| RV-ESV indexed (ml/m²) | 24.7 ± 7.9 | 24.5 ± 8.0 | 0.871 |
| RV-SV (ml) | 82.9 ± 17.8 | 83.3 ± 14.8 | 0.893 |
| RV-SV indexed (ml/m²) | 43.7 ± 6.0 | 44.3 ± 6.0 | 0.609 |
| RV-CO (l/min) | 5.8 ± 1.3 | 5.8 ± 1.1 | 0.869 |
| RV-CO / BSA (l/min/m²) | 3.1 ± 0.5 | 3.1 ± 0.6 | 0.837 |
| RA Diameter (mm) | 43.9 ± 5.2 | 43.7 ± 5.9 | 0.862 |
| **Native T1 times** |  |  |  |
| **1.5-T** | n=22 | n=21 |  |
| anterior (ms) | 1017 ± 37 | 1000 ± 54 | 0.235 |
| antero-lateral (ms) | 1001 ± 33 | 989 ± 31 | 0.190 |
| infero-lateral (ms) | 1032 ± 31 | 1022 ± 42 | 0.369 |
| inferior (ms) | 1049 ± 40 | 1058 ± 27 | 0.416 |
| infero-septal (ms) | 1057 ± 40 | 1032 ± 22 | <0.05 |
| antero-septal (ms) | 1035 ± 26 | 1012 ± 33 | <0.05 |
| **3-T** | n=19 | n=26 |  |
| anterior (ms) | 1299 ± 83 | 1246 ± 52 | <0.05 |
| antero-lateral (ms) | 1226 ± 32 | 1210 ± 33 | 0.115 |
| infero-lateral (ms) | 1224 ± 63 | 1241 ± 37 | 0.277 |
| inferior (ms) | 1242 ± 57 | 1250 ± 36 | 0.581 |
| infero-septal (ms) | 1293 ± 69 | 1250 ± 39 | <0.05 |
| antero-septal (ms) | 1293 ± 49 | 1262 ± 28 | <0.01 |

Cardiac Magnetic Resonance (CMR) Measurements: LBBB – left bundle-branch block; LV – left ventricle; ESV - end-systolic volume; SV – stroke volume; CO – cardiac output; LA – left atrium; RV – right ventricle; RA – right atrium; Comparison of native T1 times between LBBB patients and controls in 1.5-Tesla (1.5-T) and 3-Tesla MRI (3-T): Values are mean ± standard deviation or median (interquartile range).

Table S3 Correlation coefficients between QRS duration and functional and structural measurements in patients with idiopathic LBBB.

|  | **r²** | **r** | **p** |
| --- | --- | --- | --- |
| **QRS duration (ms)** |  |  |  |
| LV-ESV indexed (ml/m²) | 0.533 | 0.730 | < 0.001 |
| LV-CO indexed (l/min/m²) | 0.025 | -0.159 | 0.936 |
| RV-EDV indexed (ml/m²) | 0.359 | 0.599 | < 0.001 |
| RV-ESV indexed (ml/m²) | 0.229 | 0.479 | < 0.001 |
| RV-CO indexed (l/min/m²) | 0.003 | -0.052 | 0.869 |

Correlation between Cardiac Magnetic Resonance Measurements (CMR) and QRS duration: LBBB – left bundle-branch block; LV – left ventricle; ESV - end-systolic volume; CO – cardiac output; RV – right ventricle; EDV - end-diastolic volume; r^2^ – coefficient of determination. Pearson correlation was used to calculate linear relationships between CMR and QRS duration (p<0.05 is considered significant).

Table S4 Comparison of idiopathic LBBB Patients with isolated ventricular asynchrony and with superimposed contractile impairment.

| **CMR Measurements** | **LBBB with isolated asynchrony** (n=33) | **LBBB with superimposed hypocontractility** (n=20) | **Controls** (n=53) | **ANOVA** | **Bonferroni Post-Hoc** | | |
| --- | --- | --- | --- | --- | --- | --- | --- |
|  |  |  |  | p | LBBB with isolated asynchrony  vs.  LBBB with superimposed hypocontractility | LBBB with isolated asynchrony  vs.  Controls | LBBB with superimposed hypocontractility vs.  Controls |
| LV-ESV indexed (ml/m²) | 34.8 ± 8.9 | 45.1 ± 8.5 | 26.8 ± 5.5 | <0.001 | <0.001 | <0.001 | <0.001 |
| LV-CO indexed (l/min/m²) | 3.1 ± 0.5 | 3.0 ± 0.4 | 3.2 ± 0.7 | 0.594 | 1.000 | 0.929 | 1.000 |
| LV mass (g) | 93 ± 25 | 103 ± 30 | 88 ± 22 | 0.068 | 0.397 | 1.000 | 0.062 |
| RV-ESV indexed (ml/m²) | 21.6 ± 5.3 | 29.9 ± 8.9 | 24.5 ± 8.0 | <0.001 | <0.001 | 0.267 | <0.05 |
| RV-CO indexed (l/min/m²) | 3.0 ± 0.5 | 3.1 ± 0.5 | 3.1 ± 0.6 | 0.868 | 1.000 | 1.000 | 1.000 |
| **Native T1 times** |  |  |  |  |  | | |
| **1.5-T** | n=14 | n=8 | n=22 |  |  |  |  |
| anterior (ms) | 1006 ± 33 | 1037 ± 37 | 1000 ± 54 | 0.152 | 0.337 | 1.000 | 0.170 |
| antero-lateral (ms) | 1030 ± 25 | 1046 ± 35 | 989 ± 31 | 0.231 | 0.806 | 1.000 | 0.267 |
| infero-lateral (ms) | 1034 ± 32 | 1030 ± 29 | 1022 ± 42 | 0.651 | 1.000 | 1.000 | 1.000 |
| inferior (ms) | 1041 ± 42 | 1064 ± 36 | 1058 ± 27 | 0.227 | 0.392 | 0.457 | 1.000 |
| infero-septal (ms) | 1048 ± 32 | 1073 ± 50 | 1032 ± 22 | <0.05 | 0.241 | 0.453 | <0.05 |
| antero-septal (ms) | 1030 ± 24 | 1044 ± 30 | 1012 ± 33 | <0.05 | 0.851 | 0.254 | <0.05 |
| **3-T** | n=11 | n=8 | n=26 |  |  |  |  |
| anterior (ms) | 1285 ± 60 | 1287 ± 70 | 1246 ± 52 | 0.035 | 1.000 | 0.406 | <0.05 |
| antero-lateral (ms) | 1213 ± 25 | 1245 ± 34 | 1210 ± 33 | 0.030 | 0.103 | 1.000 | <0.05 |
| infero-lateral (ms) | 1231 ± 39 | 1216 ± 89 | 1241 ± 37 | 0.451 | 1.000 | 1.000 | 0.655 |
| inferior (ms) | 1243 ± 59 | 1242 ± 57 | 1250 ± 36 | 0.860 | 1.000 | 1.000 | 1.000 |
| infero-septal (ms) | 1281 ± 62 | 1309 ± 80 | 1250 ± 39 | 0.023 | 0.782 | 0.353 | <0.05 |
| antero-septal (ms) | 1283 ± 49 | 1307 ± 48 | 1262 ± 28 | 0.013 | 0.524 | 0.372 | <0.05 |

Comparison between the following group: isolated LBBB patients with isolated ventricular asynchrony (LBBB with isolated asynchrony), with superimposed contractile impairment (LBBB with superimposed hypocontractility) and controls in 1.5-Tesla (1.5-T) and 3-Tesla MRI (3-T): LV – left ventricle; ESV - end-systolic volume; CO – cardiac output; RV – right ventricle. Values are mean ± standard deviation. Differences between all three groups were calculated using 2-way ANOVA, Bonferroni-Posttest between all three subgroups.

1. Schulz-Menger J, Bluemke DA, Bremerich J, Flamm SD, Fogel MA, Friedrich MG, et al. Standardized image interpretation and post processing in cardiovascular magnetic resonance: Society for Cardiovascular Magnetic Resonance (SCMR) board of trustees task force on standardized post processing. *Journal of cardiovascular magnetic resonance : official journal of the Society for Cardiovascular Magnetic Resonance*. 2013 May 1;**15**:35. PubMed PMID: 23634753. Pubmed Central PMCID: PMC3695769. Epub 2013/05/03. eng.
